# Microbial-Assisted Phytoremediation of Glyphosate-Contaminated Soil by *Medicago sativa*: Biochemical and Detoxification Responses, Gene Expression, and Dissipation Kinetics

**DOI:** 10.3390/toxics14070621

**Published:** 2026-07-16

**Authors:** Ahmed A. A. Aioub, Ahmed Fayez Omar, Ahmed S. Hashem, Hosny Kesba, Sherif El-Ganainy, Wael Elmenofy, Mohamed El-Mogy, Mostafa Almaghaslah, Mustafa Shukry, Zhang Lijun, Qichun Zhang, Sarah I. Z. Abdel Wahab

**Affiliations:** 1 State Key Laboratory of Soil Pollution Control and Safety, Key Laboratory of Environment Remediation and Ecological Health, Ministry of Education, Zhejiang University, Hangzhou 310058, China; a.aioub@zu.edu.eg; 2 Plant Protection Department, Faculty of Agriculture, Zagazig University, Zagazig 44511, Egypt; 3 Pesticides Chemistry and Toxicology Department, Faculty of Agriculture, Kafrelsheikh University, Kafrelsheikh 33516, Egypt; 4 Stored Product Pests Research Department, Plant Protection Research Institute, Agricultural Research Center, Kafr El-Sheikh 33717, Egypt; 5 Department of Arid Land Agriculture, College of Agricultural and Food Sciences, King Faisal University, Al-Ahsa 31982, Saudi Arabia; salganainy@kfu.edu.sa (S.E.-G.); welmenofy@kfu.edu.sa (W.E.);; 6 Pests and Plant Diseases Unit, College of Agriculture and Food Sciences, King Faisal University, Al-Ahsa 31982, Saudi Arabia; malmghaslah@kfu.edu.sa; 7 Department of Biomedical Sciences, College of Veterinary Medicine, King Faisal University, Al-Ahsa 31982, Saudi Arabia; matta@kfu.edu.sa; 8 Pingyang Country Bureau of Agriculture and Rural Affairs, Wenzhou 325400, China

**Keywords:** decontamination, herbicide, biosurfactant, remediation, soil contamination

## Abstract

Glyphosate (GLY), one of the most extensively applied broad-spectrum herbicides worldwide, frequently contaminates soil and aquatic ecosystems, posing serious threats to human health, non-target organisms, soil microbial communities, and environmental sustainability. In the present study, phytoremediation using *Medicago sativa* (MS) was evaluated for the removal of GLY from contaminated soil under greenhouse conditions, with remediation efficiency enhanced through inoculation with two bacterial bioagents, *Bacillus* sp. h10 (BS) and *Pseudomonas aeruginosa* KZFS4 (PA). Biochemical parameters, including superoxide dismutase (SOD), catalase (CAT), hydrogen peroxide (H_2_O_2_), and malondialdehyde (MDA), together with detoxification-related gene expression, were investigated in the roots and leaves of MS exposed to GLY stress. The combined application of MS with BS + PA, followed by MS + PA and MS + BS, significantly decreased GLY residues in soil and increased GLY accumulation in plant roots and leaves after 1, 3, 7, and 10 days compared with MS treatment alone. In vitro batch equilibrium experiments demonstrated that BS and PA desorbed 33.63 and 40.56 µg g^−1^ of GLY, respectively, thereby enhancing its removal from soil. The persistence of GLY was highest in contaminated soil without treatment, exhibiting a half-life (t_1_/_2_) of 52.66 days, whereas the shortest half-life (6.69 days) was recorded in soil treated with MS combined with BS and PA relative to sterilized contaminated soil. Furthermore, inoculation with BS and PA markedly increased SOD and CAT activities in MS tissues, while significantly reducing H_2_O_2_ and MDA accumulation, indicating alleviation of oxidative stress. GLY exposure also triggered substantial upregulation of detoxification-associated genes, including cytochrome P450, glutathione S-transferases (GST), glycosyltransferases (GTs), and ABC transporters in MS. These findings demonstrate that the integration of BS and PA with phytoremediation effectively accelerates GLY dissipation and reduces pesticide-associated toxicity in contaminated soils and plants.

## 1. Introduction

Glyphosate (GLY; N-(phosphonomethyl) glycine) has been the most extensively applied broad-spectrum herbicide worldwide since its commercial introduction in 1971 [1]. The widespread adoption of GLY-resistant crops has significantly increased its global application in modern agricultural systems [2]. Glyphosate (GLY) is extensively used because of its high efficiency in controlling annual and perennial weeds in monoculture farming systems [3]. However, The excessive and repeated application of GLY has generated significant environmental concerns because of its persistence and accumulation in soil and aquatic ecosystems [4].

GLY residues have been widely detected in agricultural soils, groundwater, surface waters, wetlands, and even atmospheric samples across the globe [5]. GLY use has increased dramatically worldwide since the commercialization of GLY-resistant crops in the mid-1990s [6]. Current GLY application rates in the United States may reach 3.9 kg ha^−1^ in soybean fields and 6.6 kg ha^−1^ in corn production systems [7]. In China, GLY production exceeded 500,000 tons annually, making China the largest producer and exporter of GLY worldwide [8]. Another study also reported an increase in GLY residues in agricultural soils and surface waters due to the extensive application of GLY-based herbicides in intensive farming systems in China [9]. In Egypt, GLY-based herbicides are increasingly used for weed management in orchards and field crops, and GLY residues have been detected in agricultural soils and environmental samples under Egyptian conditions [10]. High GLY concentrations in environmental compartments may negatively affect soil fertility, microbial diversity, and ecological balance [11]. Several studies demonstrated that GLY exposure suppresses beneficial soil microorganisms and alters soil enzymatic activities involved in nutrient cycling [11,12,13]. Furthermore, GLY toxicity may induce oxidative stress, membrane damage, and metabolic disturbances in non-target organisms [14]. Therefore, developing environmentally sustainable strategies for GLY remediation has become an urgent necessity.

Phytoremediation is an approach that utilizes plants to break down, stabilize, and eliminate pollutants from the environment [15]. This technique is widely used to remove organic and heavy metal pollutants from contaminated sites [16]. The removal, accumulation, transport, and degradation of contaminants by plants are considered key mechanisms in phytoremediation technology [17]. Phytoremediation takes advantage of the plant root system’s ability to target and uptake contaminants and systemically translocate them through plant tissues, facilitating their detoxification and removal [18]. As a result, phytoremediation is an environmentally friendly approach for preventing, controlling, and cleaning up pollution.

*Medicago sativa* L. (MS) also known as alfalfa, is a perennial leguminous plant widely cultivated due to its rapid growth, high biomass production, and strong adaptability to environmental stresses [19]. The plant possesses a deep root system that enhances soil stabilization and promotes rhizosphere microbial activity involved in pollutant degradation [20]. Several studies demonstrated that MS can tolerate and accumulate various contaminants, including heavy metals, pesticides, and organic xenobiotics, making it a promising candidate for phytoremediation applications [21,22]. In addition, MS enhances the biodegradation of pollutants through plant–microbe interactions and activation of antioxidant defense systems under stress conditions [23]. Therefore, MS is considered an environmentally sustainable species for the decontamination of agricultural soils and the restoration of environmental quality [24].

Several studies have investigated the use of phytoremediation for pesticide elimination from polluted soil [25,26,27]. Nevertheless, phytoremediation has certain limitations, including the inability to extract contaminants located below the rooting depth. It is also a time-consuming process, constrained by plants’ limited capacity to accumulate high levels of xenobiotics and maintain normal growth in toxic conditions [28]. Another disadvantage is the plant’s capacity for accumulation and sensitivity to high soil contamination levels [29]. Therefore, combining plants with bioagents may enhance and accelerate the effectiveness of phytoremediation. The application of plant–microbial approaches to improve phytoremediation may represent an effective strategy [30]. Pollutant-degrading bacteria can support plant adaptation to contaminated environments by detoxifying the soil through the direct mineralization of organic contaminants [31]. In addition, compounds released by plants can stimulate the growth and activity of potential pollutant-degrading bacteria within the rhizosphere [32]. Several studies have documented the application of plant–microbe associations in the remediation of environmental pollutants [33,34,35,36,37]. Nevertheless, studies investigating herbicide removal using this approach remain limited.

Plants possess diverse defense and detoxification strategies to combat the negative effects of xenobiotic toxicity [38]. Pesticide toxicity is linked to different biochemical processes in plants [39]. An imbalance between antioxidant defenses and free radicals induces oxidative stress, resulting in the production of reactive oxygen species (ROS) that can damage plant cells [40]. The defense mechanisms of plants, including antioxidant enzymes like superoxide dismutase (SOD) and catalase (CAT), help prevent or reduce damage caused by ROS [39]. Enzymes that scavenge ROS, such as SOD, facilitate the conversion of hydrogen peroxide (H_2_O_2_) through dismutation, after which the CAT enzyme breaks down the resulting hydrogen peroxide into oxygen molecules and water [41]. In addition, they catalyze different pathways to transform ROS into more stable substances, thereby reducing the potential damage caused by pesticide-induced oxidative stress [42]. Malondialdehyde (MDA) is produced as secondary product of membrane lipid oxidation during the degradation of polyunsaturated fatty acids. Its concentration serves as an indicator of lipid peroxidation and oxidative damage [43]. Exposure to toxic substances commonly triggers ROS production and lipid peroxidation [44]. Meanwhile, a series of detoxification mechanisms has been suggested for xenobiotics. The “green-liver” system is a defense mechanism that enables plants to detoxify pesticides through three distinct phases: phase I involves the activation of toxic compounds by cytochrome P450 (Cyt-P450), phase II includes molecular conjugation by glutathione S-transferases (GSTs) and glycosyltransferases (GTs), and phase III involves the sequestration of these compounds in plant organelles or structures [45,46].

Our findings hypothesized that the phytoremediation of MS in the presence of bioagents could provide an effective, cost-efficient, and rapid method for removing GLY residues from polluted soil. This study aimed to evaluate the potential of MS and two bioagents (*Bacillus* sp. h10 (BS) and *Pseudomonas aeruginosa* KZFS4 (PA)) to removal and take up GLY-polluted soil under greenhouse conditions. Furthermore, the investigation of the antioxidant enzymes (SOD and CAT) and oxidative stress (H_2_O_2_ and MDA) in MS under GLY stress alone and with two tested agents. Moreover, the determination of the expression profile of some detoxification genes in MS under GLY stress.

## 2. Materials and Methods

### 2.1. Chemicals Compounds and Bioagents

GLY (purity ≥ 98%, China) was purchased from Sigma-Aldrich (Shanghai) Trading Co., Ltd. (Shanghai, China) China. GLY at 5 µg g^−1^ was mixed with the irrigation water and added to the soil to prevent direct contact between GLY and plant leaves. *Bacillus* sp. *h10* (BS) and *Pseudomonas aeruginosa* KZFS4 (PA) were obtained from previous studies conducted by our research group [26,47,48]. *Bacillus* sp. h10 was originally isolated from cadmium-contaminated agricultural soil in Zhejiang Province, China, and has been previously characterized for its bioremediation potential. Pseudomonas aeruginosa KZFS4 was isolated from pesticide-contaminated environmental samples collected from industrial sites in Egypt and is publicly available in the NCBI GenBank database under accession number LC599404.1. This strain was previously identified using 16S rRNA gene sequencing analyses. Both strains were selected based on their previously reported environmental resilience and bioremediation potential, which makes them suitable candidates for evaluating their role in enhancing GLY removal in the present study. Moreover, Commercial kits for the determination of CAT, SOD, MDA, and H_2_O_2_ were sourced from Sigma-Aldrich, China. For the extraction and derivatization of GLY residues, HPLC-grade acetonitrile, acetic acid, 9-fluorenylmethylchloroformate (FMOC-Cl), sodium tetraborate decahydrate (borate buffer), and glycine were purchased from Sigma-Aldrich, China.

### 2.2. Greenhouse Experimental Design

A greenhouse experiment was performed under natural light conditions at temperatures of 25–27 °C and relative humidity levels of 66–69% to assess the influence of MS, either alone or in combination with the tested bacterial strains, on the removal of GLY residues from contaminated soil. The soil used in this study was collected from a site in Giza, Egypt. The soil (clay loam) was first air-dried and then sieved before experimental use. Its physicochemical properties included 1.82% organic matter, pH 7.1, and electrical conductivity of 2.28 S m^−1^. The prepared soil was subsequently distributed into pots, with each pot containing 500 g of soil. Seeds of MS were obtained from the Agricultural Research Center, Egypt. A completely randomized design was applied to the experimental pots, including eight treatments: (T1) GLY-contaminated sterilized soil (GCSS) without *M. sativa* (MS), (T2) GLY-contaminated soil (GCS) without MS, (T3) GCSS with MS only (GCSS + MS), (T4) GCS with MS only (GCS + MS), (T5) GCS with MS and amended with *Bacillus* sp. h10 (GCS + MS + BS) at 10^7^ CFU mL^−1^, (T6) GCS with MS and amended with *P. aeruginosa* KZFS4 (GCS + MS + PA) at at 10^7^ CFU mL^−1^, (T7) GCS with MS and amended with *Bacillus* sp. h10, and *P. aeruginosa* KZFS4 (GCS + MS + BS + PA), (T8) uncontaminated soil with MS (S + MS). A total of 96 pots were prepared for all experimental treatments. Each treatment consisted of three independent biological replicates (three separate pots per sampling time), and each pot was considered an independent experimental unit. All measurements were performed on samples collected from independent pots. Each pot was sown with five MS seeds. Following germination, the seedlings were carefully thinned to retain a single plant in each pot. Samples of plants were harvested from treated soil at 1, 3, 7, and 10 days after exposure for further analysis. After careful dissection, the plants were divided into roots and leaves. The roots were washed under running tap water for 3 min and then dried. GLY residue analysis was performed using 10 g of soil samples and 4 g of both root and leaf tissues.

Based on the method described by Romeh [49], the GLY removal percentage after 1, 3, 7, and 10 days of exposure was determined according to the following equation:

(1) The percentage of removal (%) = (C_0_ − C_1_)/C_0_ × 100,

C_0_: initial GLY concentration in soil and C_1_: GLY concentration.

### 2.3. In Vitro Evaluation of GLY Adsorption–Desorption by Bioagents

An in vitro experiment was carried out to investigate the equilibrium adsorption of GLY and to evaluate the efficiency of the two tested bioagents in enhancing GLY removal from contaminated soil. The BA and PA at 10^7^ CFU mL^−1^ were separately introduced into 100 mL flasks, whereas distilled water was applied as the control treatment. The conical flasks were then treated with GLY at 5 µg/mL. Each flask was adjusted to a final volume of 20 mL. Subsequently, each flask received 1 g of soil, and the resulting mixtures were incubated with shaking for 3 h. The suspensions obtained were stored at 27 °C for 24 h, with all treatments conducted in triplicate. The samples were centrifuged at 15,000 rpm for 10 min, and the supernatants were analyzed by HPLC to determine GLY concentration. The adsorption capacity of GLY was determined by subtracting the equilibrium concentration from the initial concentration, as described by Aioub et al. [50].

(2) x/m = (Co − Ce)V/W,

whereas x/m denotes the GLY in soil concentration (µg g^−1^), C_0_ is the initial GLY concentration (µg mL^−1^), Ce is the equilibrium GLY concentration (µg mL^−1^), V represents the volume of the solution, and W refers to the weight of the soil sample.

### 2.4. GLY Residue Analysis and Kinetics

The QuEChERS method was used to extract the GLY residue from the tested samples as mentioned by Lehotay et al. [51]. Soil (8 g) and MS root and leaf tissues (5 g) were added to a 50 mL centrifuge tube and mixed with 10 mL of acetonitrile containing 1% acetic acid. The mixture was shaken vigorously for 1 min, afterwards, 6 g MgSO_4_, 1.5 g NaCl, and 1 g C_6_H_5_Na_3_O_7_·2H_2_O were added. After adding the salts, each tube was promptly shaken. After being vigorously agitated for 1 min, the mixtures were subjected to centrifugation at 4000 rpm for 5 min. One milliliter of the supernatant was subjected to clean-up using a tube containing primary secondary amine (PSA), MgSO_4_, graphitized carbon black (GCB), and C_18_. Following 1 min of vigorous shaking, the tubes were centrifuged at 4000 rpm for 5 min. GLY in the cleaned extract was derivatized with FMOC-Cl. A 500 µL aliquot of the cleaned supernatant was mixed with 500 µL of 0.1 M borate buffer (pH 9.0) and 500 µL of 5 mM FMOC-Cl solution in acetonitrile. The mixture was vortexed and allowed to react in the dark at room temperature for 30 min. After derivatization, the reaction was quenched by adding 50 µL of 0.1 M glycine solution, and the sample was filtered through a 0.22 µm PTFE syringe filter into an HPLC vial for analysis. Quantification of derivatized GLY residues was performed using a high-performance HPLC-UV. Isocratic HPLC analysis was carried out using a C18 column (e.g., 250 mm × 4.6 mm, 5 µm particle size) maintained at 25 °C. The mobile phase consisted of 60% acetonitrile and 40% water, containing 0.1% acetic acid, and was delivered at a flow rate of 1.0 mL min^−1^. Detection of the FMOC-derivatized GLY was performed at 254 nm. The retention time for derivatized GLY was approximately 7.3 min, with a total chromatographic run time of 20 min.

### 2.5. Method Validation

The analytical method was validated in terms of linearity, matrix effects, precision, accuracy, limits of detection (LOD), and limits of quantification (LOQ). Calibration curves constructed using matrix-matched standards showed excellent linearity with R^2^ = 0.9996, confirming the suitability of the method for quantitative analysis (Figure S1). Matrix effects were evaluated by comparing calibration slopes in solvent and matrix extracts (soil, root, and leaves). Precision was assessed as repeatability (intra-day, *n* = 3), expressed as relative standard deviation (RSD%), while accuracy was evaluated through recovery experiments at three spiking levels (0.05, 0.1, and 0.5 µg g^−1^) (Table S1). The LOD and LOQ were calculated based on the standard deviation of the response and the slope of the calibration curve according to Thomsen et al. [52], the LOQ and LOD values were calculated as follows:

(3) LOQ = 10S_0_/b, and LOD = 3.3S_0_/b,

In this equation, S_0_ refers to the standard deviation of the calibration curve, whereas b represents the slope of the calibration line. The LOQ and LOD values for GLY were 0.58 µg g^−1^ and 0.19 µg g^−1^, respectively. Recovery values ranged from 93.33 to 94.74% in soil, 89.05–91.69% in roots, and 90.55–93.11% in leaves, indicating satisfactory method accuracy. The representative HPLC chromatograms are shown in the Supplementary Information (Figure S2). Using the equations from [53], the degradation rate (K) and half-life (t_1/2_) were calculated as follows:

(4) The degradation rate (K) = 2.303 × slope,

(5) Half - life value(t_1/2_) = 0.693K^−1^,

### 2.6. Biochemical Stress Ndicators

After 1, 3, 7, and 10 days of GLY exposure, 100 mg of MS tissues from treatments 4–8 were harvested and homogenized at 4 °C with liquid nitrogen. Samples were collected from independent biological replicates (n = 3 per treatment per time point). The SOD activity was measured at 560 nm by a protocol according to Giannopolitis and Ries [54]. The activity of CAT was measured following the procedure reported by Cakmak and Marschner [55], and H_2_O_2_ reduction was assessed by recording the decline in absorbance at 240 nm for 1 min. The H_2_O_2_ level was assessed following the procedure of Velikova et al. [56] and measured at a wavelength of 290 nm. The level of MDA accumulation in the tissues was determined by measuring absorbance at 532 and 600 nm following the procedure reported by Hodges et al. [57].

### 2.7. qRT-PCR Analysis of Detoxification Gene Expression

Total RNA was extracted from MS roots and leaves exposed to GLY stress and control conditions after 7 days of the experiment, following the method described by Zhang et al. [58] and Li et al. [59]. cDNA synthesis was conducted using a RevertAid First Strand cDNA Synthesis Kit. Primers for four target genes were designed using Primer 5, and their sequences are listed in Table 1. The actin gene was employed as the internal control, and qRT-PCR was carried out using SYBR Premix Ex Taq™ II on a LightCycler 480 II system. Thermal cycling conditions were set at 95 °C for 30 s, followed by 40 cycles of denaturation at 95 °C for 5 s and annealing/extension at 60 °C for 20 s; relative gene expression was calculated using the 2^−ΔΔCt^ method [60]. Gene expression kits were purchased from Sigma-Aldrich, China. Biological replicates were obtained from independent plants grown in separate pots (n = 3 per treatment), and each biological replicate was analyzed independently.

### 2.8. Statistical Analysis

One-way ANOVA was used for statistical analysis, followed by Tukey’s post hoc test in GraphPad Prism 10 to compare treatment means (mean ± SD). Differences were considered statistically significant at *p* < 0.05. Relative gene expression levels were calculated using the 2^−ΔΔCt^ method.

## 3. Results

### 3.1. Effect of MS and Bioagents on GLY Removal

Across the different treatments, GLY concentrations in soil were monitored over a period of 1–10 days (Table 2) to evaluate the capacity of MS to remove GLY. The assessment also aimed to determine the role of various surfactants in enhancing GLY bioavailability, increasing microbial abundance, improving MS performance, and promoting synergistic interactions between MS and microorganisms, ultimately facilitating GLY removal in soil. After 7 days of exposure, GLY removal percentages in the control (T_1_) and the different treatments were compared. The combined application of MS and microorganisms (T_4_–T_1_) achieved the highest efficiency for GLY removal, increasing removal by 31.20% compared with natural attenuation. This was followed by MS alone (T_3_–T_1_) and microorganisms alone (T_2_–T_1_), which increased removal by 22.80% and 15.00%, respectively. In addition, PA (T_6_) showed a greater effect on GLY uptake than BS (T_5_). The combined inoculation of PA and BS further improved GLY removal, achieving an increase of 20.40% after 7 days.

### 3.2. Fate of GLY in Soil and Plant Tissues Under Different Treatments

**Figure 1:**
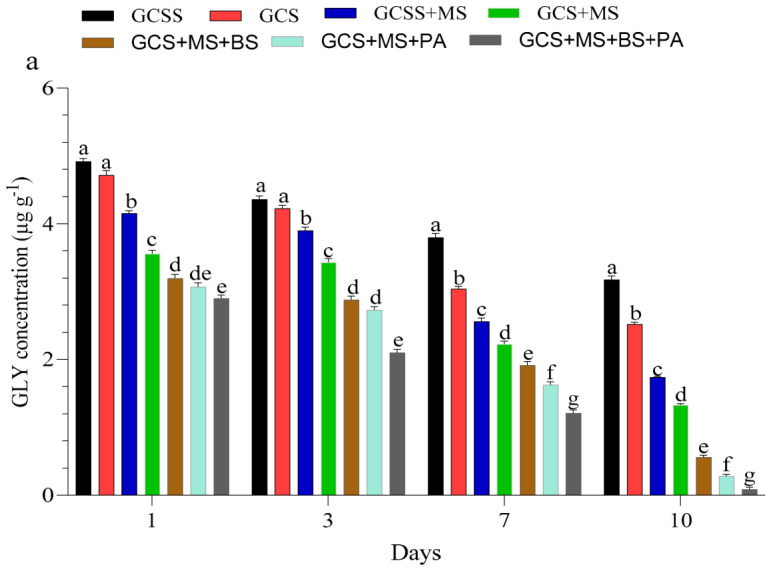

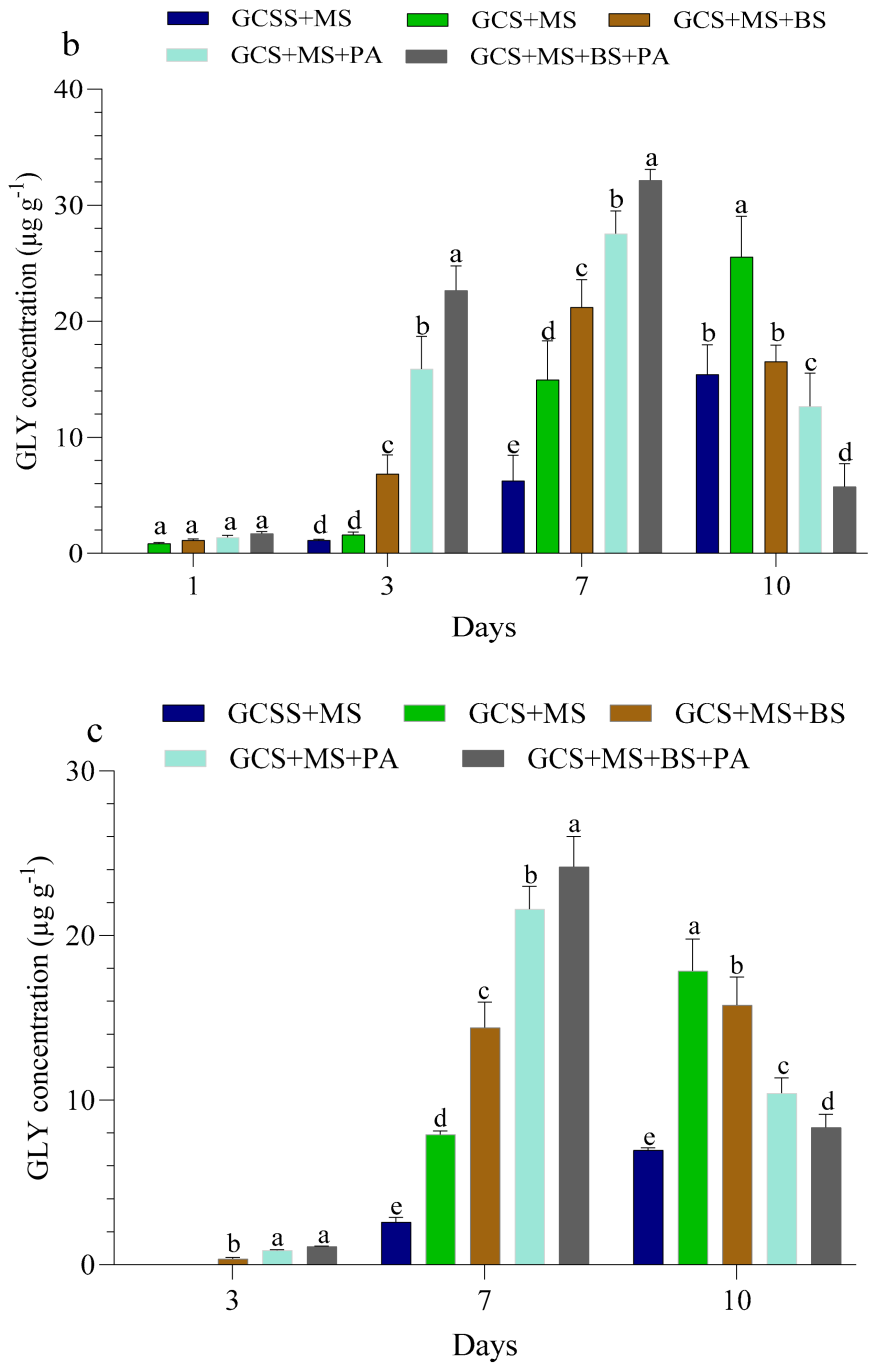
Effectiveness of the two tested agents in enhancing phytoremediation of glyphosate (GLY)-contaminated soil using MS over a 10-day experimental period. GLY concentrations in soil (**a**), roots (**b**), and leaves (**c**) are presented. Values are reported as mean ± standard deviation (n = 3). Bars with different letters differ significantly (*p* < 0.05) according to statistical analysis.

The distribution of GLY in soil and plant tissues (roots and leaves) over time is shown in Figure 1. The results indicated that all treatments involving plants contributed substantially to GLY removal. The combined application of MS with the tested bacterial strains reduced GLY levels in soil while simultaneously enhancing its accumulation in roots and leaves. PA exhibited a pronounced synergistic effect on both uptake and translocation of GLY. Overall, MS amended with PA showed greater phytoremediation efficiency than BS, leading to almost complete elimination of GLY from contaminated soil during the experimental period.

In MS-only treatments, soil GLY concentrations ranged from 8.24 to 3.16 µg g^−1^ over 10 days, whereas in the control soil they ranged from 3.56 to 1.33 µg g^−1^. After 3 days, GLY levels in soil treated with MS and amended with BS, PA, and BS + PA were 2.87, 2.73, and 2.10 µg g^−1^, respectively, compared with 3.43 µg g^−1^ in MS alone. By day 10, soil GLY concentrations in MS combined with BS, PA, and BS + PA declined to 0.56, 0.28, and 0.09 µg g^−1^, respectively, while the control soil recorded 1.33 µg g^−1^, with significant differences observed (*p* < 0.05) (Figure 1a).

The tested bacterial inoculants (BS, PA, and their combination) also enhanced GLY uptake and translocation in MS roots. At day 3, significant accumulation was observed in roots (6.86, 15.89, and 22.65 µg g^−1^), showing differences compared with MS alone (1.59 µg g^−1^). By day 10, root GLY concentrations in the BS, PA, and BS + PA treatments were 16.53, 12.67, and 5.75 µg g^−1^, respectively, compared with 25.54 µg g^−1^ in MS alone (Figure 1b).

In leaves, GLY accumulation and movement were significantly enhanced (*p* < 0.05) at day 7 in the presence of BS, PA, and BS + PA, with increases of approximately 1.82-, 2.73-, and 3.05-fold relative to MS alone. However, by day 10, GLY levels in leaves decreased to approximately 88%, 58%, and 47% in the BS, PA, and BS + PA treatments, respectively, compared with MS alone (Figure 1c).

**Figure 2:**
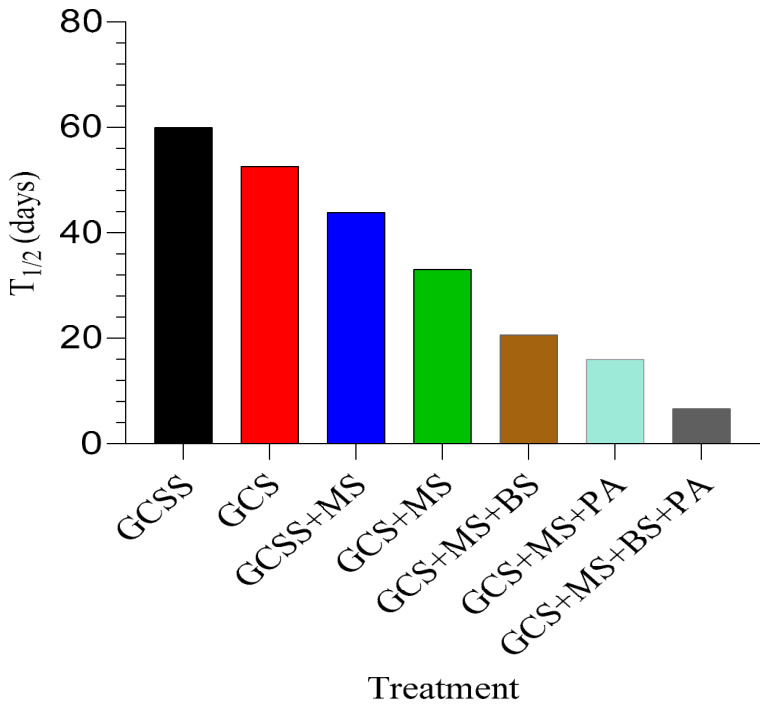
Changes in the half-life (t_1/2_) of glyphosate (GLY) under different soil treatments.

A first-order reaction can adequately describe the removal of GLY from the soil. According to our data in Figure 2, the t_1/2_ of GLY for all treatments was as follows: GCSS > GCS > GCSS + MS > GCS + MS > GCS + MS + BS > GCS + MS + PA > GCS + MS + BS + PA. Table S2 shows the kinetic fitting parameters and first-order dissipation modeling of GLY in soil.

### 3.3. Agents Improved the GLY Recovery

**Figure 3:**
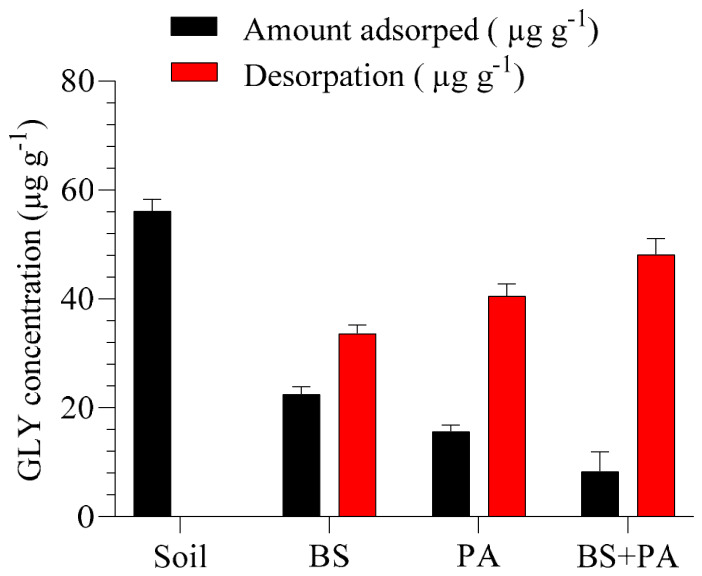
Effect of the tested agents on glyphosate (GLY) recovery from soil after 1 day of exposure. Values are shown as mean ± standard deviation based on three replicates (n = 3). Amount adsorbed is the amount of GLY retained in the soil phase. Desorption is GLY in the water phase.

An in vitro batch equilibrium experiment was conducted to evaluate the ability of the tested bioagents to influence GLY partitioning between soil and the aqueous phase. The concentrations reported represent the amount of GLY recovered in the supernatant (aqueous phase), which reflects GLY released (desorbed) from the soil matrix into solution. Distilled water was used as the control treatment. As shown in Figure 3, PA induced a higher release of GLY into the aqueous phase compared with BS, indicating a stronger effect on GLY mobilization from soil particles. After 1 day, the amounts of GLY recovered in the liquid phase were 33.63 and 40.56 µg g^−1^ for BS and PA, respectively. The combined bacterial treatment resulted in a GLY recovery of 48.16 µg g^−1^, compared with 56.16 µg g^−1^ in the untreated control soil.

### 3.4. Effects of the Tested Agents on the Physiological Parameters of MS Under GLY Stress

**Figure 4:**
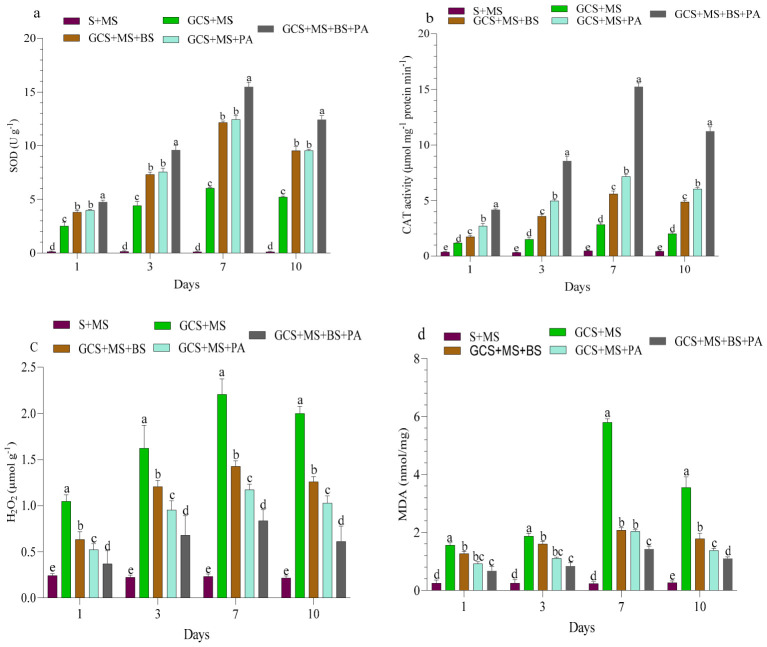
Levels of superoxide dismutase (SOD) (**a**), catalase (CAT) (**b**), hydrogen peroxide (H_2_O_2_) (**c**), and malondialdehyde (MDA) (**d**) in *Medicago sativa* (MS) roots treated with BS and PA in GLY-contaminated soil over a 1–10 day exposure period. Values are shown as mean ± standard deviation based on three replicates (n = 3). Bars with different letters differ significantly (*p* < 0.05) according to statistical analysis.

**Figure 5:**
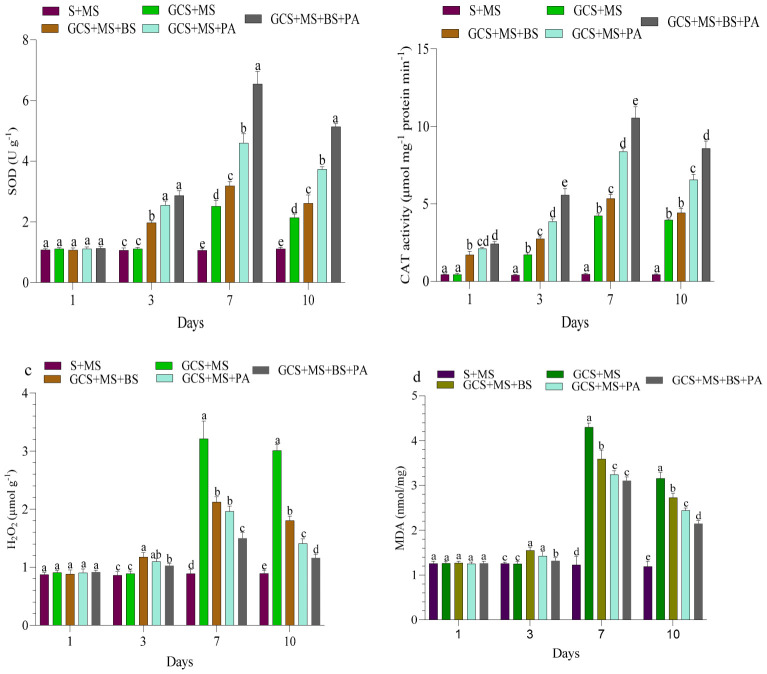
Changes in antioxidant enzyme activities and oxidative stress markers in leaves of *Medicago sativa* (MS) treated with *Bacillus* sp. h10 (BS) and *Pseudomonas aeruginosa* KZFS4 (PA) under glyphosate (GLY) contamination over a 1–10 day exposure period. Parameters include superoxide dismutase (SOD) (**a**), catalase (CAT) (**b**), hydrogen peroxide (H_2_O_2_) (**c**), and malondialdehyde (MDA) (**d**), indicating systemic oxidative stress responses in aerial plant tissues. Values are expressed as mean ± standard deviation (n = 3). Different letters indicate significant differences (*p* < 0.05).

The SOD and CAT levels in MS roots gradually increased (*p* < 0.05) in all treatments under GLY stress until the seventh day of treatment, then reduced toward the end of the study (Figure 4a,b). The SOD and CAT activities reached 12.45 U g^−1^ and 7.17 μmol mg^−1^ protein min^−1^ in the GCS + MS + PA treatment at 7 days of exposure, followed by 12.17 U g^−1^ and 5.60 μmol mg^−1^ protein min^−1^ in the GCS + MS + BS treatment compared with 6.03 U g^−1^ and 2.84 μmol mg^−1^ protein min^−1^ in GCS + MS, respectively. Meanwhile, the combination of PA + BS recorded 15.48 U g^−1^ and 15.24 μmol mg^−1^ protein min^−1^, respectively. On the tenth day of the experiment, the SOD and CAT activity were recorded at 12.43 U g^−1^ and 11.23 μmol mg^−1^ protein min^−1^ with GCS + MS + BS + PA treatment, followed by 9.55 U g^−1^ and 6.05 μmol mg^−1^ protein min^−1^ with GCS + MS + PA, 9.54 U g^−1^ and 4.88 μmol mg^−1^ protein min^−1^ with GCS + MS + BS, compared with GCS + MS, respectively. In MS leaves, the SOD and CAT activity under GLY stress after 7 and 10 days were achieved 4.60, 3.73 U g^−1^ and 8.38, 6.55 μmol mg^−1^ protein min^−1^ in GCS + MS + PA and 3.19, 2.61 U g^−1^ and 5.34, 4.42 μmol mg^−1^ protein min^−1^ in GCS + MS + BS treatment. Whilst the activities of the above enzymes were 6.55, 5.14 U g^−1^ and 10.55, 8.57 μmol mg^−1^ protein min^−1^ in GCS + MS + BS + PA treatment compared with GCS + MS treatment, respectively (Figure 5a,b).

Changes in H_2_O_2_ and MDA contents were significantly reduced (*p* < 0.05) in the MS roots and leaves plus BS and PA compared with MS alone under GLY stress (Figure 4 and Figure 5c,d). H_2_O_2_ content on the seventh day reached 1.42, 1.17 µmol g^−1^ in roots and 2.12, 1.96 µmol g^−1^ in leaves with GCS + MS + BS and GCS + MS + PA treatments, respectively. Whereas the H_2_O_2_ content was reduced to 0.83 and 1.5 µmol g^−1^ in GCS + MS + BS + PA treatment compared with GCS + MS treatment alone (2.20 and 3.21 µmol g^−1^), respectively. MDA content after 7 days of stress in the MS roots and leaves reached 5.80 and 4.30 nmol/mg with GCS + MS treatment, 2.08, and 3.59 nmol/mg with GCS + MS + BS treatment, 2.04, and 3.24 nmol/mg with GCS + MS + PA treatment, 1.42, and 3.10 nmol/mg with GCS + MS + BS + PA treatment, respectively.

### 3.5. Differential Expression of Detoxification Genes in MS Under GLY Stress Conditions

**Figure 6:**
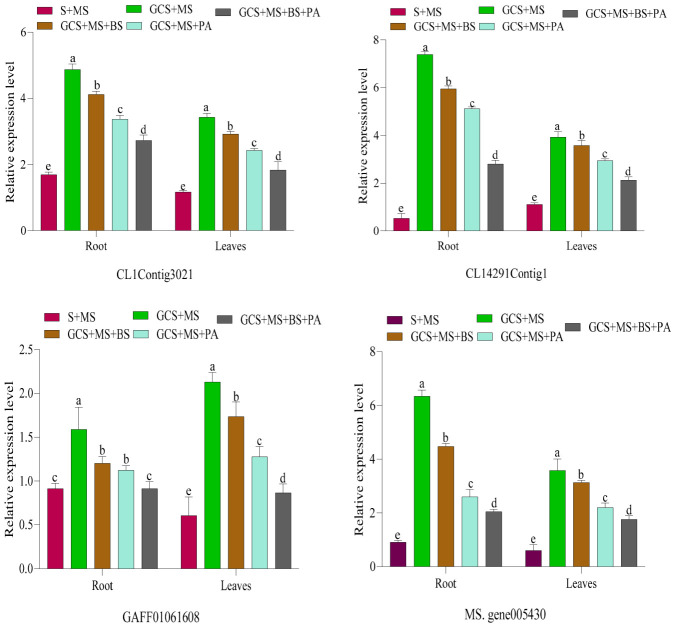
Relative expression levels of key detoxification-related genes in roots and leaves of *Medicago sativa* (MS) in response to glyphosate (GLY) exposure after 7 days. Genes analyzed include Cyt-P450, GST, GTs, and ABC transporters, which are associated with herbicide detoxification and stress adaptation. Values are expressed as mean ± standard deviation (n = 3). Different letters indicate statistically significant differences among treatments (*p* < 0.05).

The expression profiles of four detoxification genes in MS, including Cyt-P450, GST, GT, and ABC transporters, were upregulated under GLY stress after 7 days of exposure (Figure 6). The mRNA levels of GCS + MS treatment in the roots and leaves were upregulated to 4.87 and 3.42 -fold for CL1Contig3021, 7.39 and 3.93-fold for CL14291Contig1, 1.59 and 2.13-fold for GAFF01061608, 6.34 and 3.58-fold for MS. gene005430, respectively. The mRNA levels in MS root and leaves recorded 4.47 and 3.13-fold for MS. gene005430, 4.12 and 2.92-fold for CL1Contig3021, 5.95 and 3.58-fold for CL14291Contig1, 1.20 and 1.73-fold for GAFF01061608 under GCS + MS + BS treatment. While, the GCS + MS + PA treatments recorded 3.37 and 2.42-fold for CL1Contig3021, 5.12 and 2.95 for CL14291Contig1, 1.12 and 1.27-fold for GAFF01061608, and 2.60 and 2.20-fold for MS. gene005430 compared with S + MS treatment, respectively.

## 4. Discussion

Phytoremediation is a widely used bioremediation method that effectively addresses a range of pesticide contaminants [15,26]. Our results showed that the combination of MS (T_4_–T_1_) and microorganisms was the best treatment for GLY removal in soil. Phytoremediation appears to be enhanced through degradation processes driven by the combined interactions of microorganisms and plants in the rhizosphere [61]. This finding is consistent with the studies of Gurska et al. [62] and Chen et al. [63], who demonstrated that microbial-assisted phytoremediation is a promising technology for the remediation of organic pollutant-contaminated soils. In addition, these bacteria can assist plant adaptation under contaminated conditions and promote soil detoxification through the direct mineralization of organic pollutants [64]. Furthermore, plant exudates play a significant role in enhancing the activity and abundance of pollutant-degrading bacteria in the rhizosphere surrounding plant roots [65]. Also, root exudates can facilitate bioremediation processes by enhancing the growth and metabolic activity of microorganisms in the rhizosphere [66].

Our results demonstrated that MS is capable of absorbing GLY from soil and plays a key role in enhancing its removal from contaminated soil. This ability is demonstrated because of the high biomass, extensive root system, and rapid growth of MS may contribute to its phytoremediation potential [67]. In addition, bacterial inoculation enhanced GLY accumulation in plant tissues, suggesting that rhizobacteria improved herbicide bioavailability and uptake efficiency [68]. Also, the ability of MS to uptake and tolerate herbicides from contaminated soil may be associated with the production of several bioactive compounds, including glutathione, phytochelatins, phenolic compounds, flavonoids, and low-molecular-weight thiols that function as antioxidants and metal chelators, thereby neutralizing reactive oxygen species and enhancing detoxification [69]. These observations corroborate the findings of Chen et al. [70] who reported that MS is commonly used in phytoremediation due to its high biomass yield, tolerance to potentially toxic elements, and effective uptake capacity. In addition, MS enhanced remediation of polycyclic aromatic hydrocarbon-contaminated soils [71]. Sui et al. [72] showed physiological and molecular responses of MS under atrazine herbicide stress, supporting its tolerance and remediation potential. Interestingly, Several factors influence pesticide sorption in soil and its translocation within plant tissues, including soil characteristics, physicochemical properties of the pesticide (such as lipophilicity or hydrophilicity), water solubility, and parameters such as log K_ow_ and log K_oc_, values [73]. Turgut [74] demonstrated that the uptake and translocation of organic compounds depend on factors including hydrophobicity (lipophilicity), solubility, polarity, molecular weight, plant species, and environmental conditions. The uptake of GLY in soil and its translocation within MS roots and leaves may be attributed to its physicochemical properties, including its lipophilic nature, a water solubility of 10–12 g/L, as well as log K_oc_ (3.2–3.4) and log K_ow_ values (2.9–4.5) [75]. Consequently, All the above studies prove that MS could play a key role in removing pesticides from contaminated soil. However, there is a need to accelerate the phytoremediation process, as it typically takes an extended period to degrade xenobiotics in contaminated soil [76].

The application of MS in combination with BS and PA strains led to a reduction in GLY levels in soil over the 1–10-day experimental period, alongside increased accumulation in plant roots and leaves. The use of BS and PA in contaminated soil under a batch equilibrium system enhanced GLY removal compared with soil without treatment. Our data also showed that the residue half-life value of GLY in soil with PA and BS strains was less than in other treatments. This phenomenon can be explained by a microbial degradation mechanism consisting of three main stages [77]. In the first stage, the adsorption process occurs as GLY interacts with microbial cell membrane surfaces under dynamic equilibrium, playing an essential role in the system. In the second stage, the compound is transported across the cell membrane, where its uptake rate and efficiency are determined via its molecular structure. In the final stage, Enzymatic reactions within the cell rapidly convert GLY into its metabolites. Góngora-Echeverría et al. [78], previously reported that bacterial bioremediation effectively detoxifies accumulated pesticide residues in the environment. Moreover, *Bacillus subtilis* strain Bs-15 was able to significantly degrade GLY in soil, reaching up to ~67–72% removal within 96 h, demonstrating strong bioremediation potential [79]. Zhao et al. [80] demonstrated that *Pseudomonas* spp. strains (GA07, GA09, and GC04) were shown to utilize GLY as a carbon source and increased soil GLY removal by 2–3 times compared with non-inoculated controls. Likewise, *Pseudomonas alcaligenes* Z1–1 degraded GLY via enzymatic pathways, including glyphosate oxidoreductase and C–P lyase, confirming a multi-pathway biodegradation mechanism [81]. A study showed that GLY degradation in agricultural soils is mainly driven by microbial activity, with half-lives ranging from 4 to 19 days depending on soil microbial composition [82]. Yang et al. [83] reported that GLY dissipation in loess soil is rapid in the early phase, with an initial half-life of approximately 3.5 days, strongly influenced by microbial degradation processes. In addition, *Bacillus albus* strain F9D can efficiently degrade GLY in soil, achieving up to ~78% removal within 5 days, confirming its strong bioremediation potential [84].

Several studies have shown that plants have evolved an advanced antioxidant defense system that depends on SOD and CAT to counteract ROS [85,86]. Improved stress tolerance in plants is associated with exposure to different stress factors, particularly through the activation of antioxidant enzyme systems [27]. Our results revealed a significant increase in SOD and CAT activities and a concurrent reduction in MDA and H_2_O_2_ contents in MS roots and leaves under GLY stress when inoculated with the two bacterial strains (BS and PA), compared with MS alone. Enhanced activities of SOD and CAT were observed. The SOD enzyme plays an essential role in antioxidant defense by catalyzing the transformation of superoxide radicals into H_2_O_2_ during oxidative stress [87]. It is crucial for safeguarding cells from lipid peroxidation [88]. The antioxidant enzyme SOD is highly responsive to even small shifts in the cell’s redox balance, with its levels increasing in reaction to heightened cellular oxidative stress [89]. Catalase (CAT) functions as an essential antioxidant enzyme, shielding cells from harm caused by free radicals [90]. After SOD converts superoxide radicals into oxygen and H_2_O_2_, the CAT enzyme catalyzes the decomposition of hydrogen peroxide into water and oxygen [91]. Notably, Among the most important cellular biomolecules are unsaturated fatty acids, which are particularly susceptible to oxidation by free radicals and ROS, leading to lipid peroxidation [92]. Lipid peroxidation is a chain reaction driven by free radicals, which are initiated by an initial free radical that generates secondary radicals. These secondary radicals can then react with polyunsaturated fatty acids in cell membranes, which contain two or more double bonds, leading to the formation of lipid peroxides [93]. Interestingly, lipid peroxidation induced by xenobiotics occurs when ROS removes hydrogen atoms from fatty acids, leading to the formation of lipid radicals [94]. This triggers a chain reaction that produces short-chain alkanes and aldehydic acids, which damage the lipid structure [95]. Therefore, MDA is a primary byproduct of lipid peroxidation that disrupts membrane permeability and interferes with normal cellular functions [96]. These results are consistent with previous studies showing that bacterial inoculation can improve plant tolerance to xenobiotics via improving antioxidant enzymes activities [97]. Aioub, Fahmy, Ammar, Maher, Ismail, Yue, Zhang and Abdel-Wahab [26] found PA enhanced the SOD activity and decrease H_2_O_2_ and MDA contents in *Mentha piperita* under chlorpyrifos stress. Likewise, *Bacillus subtilis* increased production of antioxidants and decreased the oxidative stress of *P. major* in response to deltamethrin stress [15]. Likewise, *B. subtilis* reduced MDA levels and boosted antioxidant enzyme activity in MS under cadmium stress [98].

Our results indicated that the expressions of selected genes, including Cyt-P450, GST GT, and ABC transporter genes in MS were upregulated under GLY stress compared with untreated MS. The upregulation of tested genes under GLY stress may be due to the detoxification and removal processes of xenobiotics, which are typically divided into three phases: Phase I involves the activation of toxic compounds by Cyt-P450, Phase II consists of plant molecular conjugation through enzymes like GSTs and GTs, and Phase III entails the sequestration of these compounds in plant organelles or structures [45]. Our findings of our study correspond with those reported by GLY exposure, which induces strong transcriptional activation of Cyt-p450 genes, indicating their key role in oxidative metabolism of herbicide stress in plants [99]. Moreover, atrazine exposure induces formation of glutathione-, sugar-, and amino acid-conjugated metabolites, demonstrating activation of GST- and GT-mediated detoxification pathways in MS [100]. Furthermore, transcriptomic analysis of MS under herbicide stress revealed significant regulation of Cyt-p450, GST, GT, and transporter-related genes involved in xenobiotic detoxification processes [59]. Zhang, Lu, Zhang, Lu and Yang [58] demonstrated that exposure of MS to atrazine stress significantly altered the transcriptome, including upregulation of genes involved in detoxification pathways such as Cyt-p450, GST, GS, and ABC transporters, indicating activation of Phase I–III xenobiotic metabolism. Likewise, atrazine stress in MS caused significant modulation of detoxification-related gene expression, such as Cyt-p450, GST, GT, and ABC transporter families, suggesting their key roles in oxidative transformation, conjugation, and cellular compartmentalization of xenobiotics under stress conditions [101].

## 5. Conclusions

This study presents and assesses three integrated strategies aimed at improving the phytoremediation potential of *Medicago satvia* (MS) in soils contaminated with GLY. The first strategy demonstrated that the combined application of *Bacillus* sp. h10 (BS) and *Pseudomonas aeruginosa* KZFS4 (PA) significantly promoted GLY removal and its movement within plant tissues. The second approach showed that both BS and PA effectively regulated antioxidant defense enzymes and oxidative stress indicators in MS, with these responses being closely associated with GLY residue accumulation. The third strategy revealed that GLY stress markedly induced the expression of several detoxification-associated genes, including Cyt-P450, GST, GTs, and ABC transporters. The results highlight the promising role of combining biological and synthetic amendments with phytoremediation as an economical and environmentally sustainable strategy for reducing pesticide contamination in soils. Our findings provide mechanistic evidence supporting the integration of microbial inoculation with phytoremediation for improved pesticide-contaminated soil management. Although these results are promising, they were obtained under short-term greenhouse conditions, and further validation under field conditions is required to confirm their stability and practical applicability at larger spatial and temporal scales.

## Figures and Tables

**Table 1:** Primers for validating gene expression in *Medicago sativa*.

Genes	Forward-Primer	Reverse-Primer	Cycles
CL1Contig3021	TCAATTCCAAAGCCAATTATCC	TTATGCAGGCATTTCCCAAC	40
CL14291Contig1	ATGGTATTCTTCCGCTCTTCATC	ACAGTACCCTTTGCTTCCTGTTG	40
GAFF01061608	CTTGCTGGCTCAAATTCTACGA	AGAACTCTGCCTCGCTTATTGC	40
MS. gene005430	GCAAGTCAAGTAGCGAACGA	GAGCCATGAAGCCGATTGAG	40
MtEF1α	ATTCCAAAGGCGGCTGCATA	CTTTGCTTGGTGCTGTTTAGATGG	40

**Table 2:** Contribution of MS and surfactants that increase the GLY bioavailability in soil. Different letters indicate significant differences among treatments according to Tukey’s test (*p* < 0.05).

Days	Treatment	GLY (µg g^−1^)	% Removal	% Contribution	
1	T_1_: GCSS	4.92 a	1.60	-	0.0
	T_2_: GCS	4.71 b	5.80	microorganisms (T_2_–T_1_)	4.20
	T_3_: GCSS + MS	4.16 c	16.80	MS (T_3_–T_1_)	15.20
	T_4_: GCS + MS	3.56 d	28.80	Combination (T_4_–T_1_)	27.20
	T_5_: GCS + MS + BS	3.19 g	36.20	BS (T_5_–T_4_)	7.40
	T_6_: GCS + MS + PA	3.07 e	38.60	PA (T_6_–T_4_)	9.80
	T_7_: GCS + MS + BS + PA	2.90 f	42.00	BS + PA (T_7_–T_4_)	13.2
3	T_1_: GCSS	4.36 a	12.80	-	0.0
	T_2_: GCS	4.23 b	15.40	microorganisms (T_2_–T_1_)	2.60
	T_3_: GCSS + MS	3.95 c	21.00	MS (T_3_–T_1_)	8.20
	T_4_: GCS + MS	3.43 d	31.40	Combination (T_4_–T_1_)	18.60
	T_5_: GCS + MS + BS	2.87 f	42.60	BS (T_5_–T_4_)	11.20
	T_6_: GCS + MS + PA	2.73 e	45.40	PA (T_6_–T_4_)	14.00
	T_7_: GCS + MS + BS + PA	2.10 e	58.00	BS + PA (T_7_–T_4_)	26.60
7	T_1_: GCSS	3.79 a	24.20	-	0.0
	T_2_: GCS	3.04 b	39.20	microorganisms (T_2_–T_1_)	15.00
	T_3_: GCSS + MS	2.65 b	47.00	MS (T_3_–T_1_)	22.80
	T_4_: GCS + MS	2.23 c	55.40	Combination (T_4_–T_1_)	31.20
	T_5_: GCS + MS +BS	1.92 g	61.60	BS (T_5_–T_4_)	6.20
	T_6_: GCS + MS +PA	1.63 e	67.40	PA (T_6_–T_4_)	12.00
	T_7_: GCS + MS + BS + PA	1.21 f	75.80	BS + PA (T_7_–T_4_)	20.40
10	T_1_: GCSS	3.18 a	36.40		0.0
	T_2_: GCS	2.52 b	49.60	microorganisms (T_2_–T_1_)	13.20
	T_3_: GCSS + MS	1.74 b	65.20	MS (T_3_–T_1_)	28.80
	T_4_: GCS + MS	1.33 c	73.40	Combination (T_4_–T_1_)	37.00
	T_5_: GCS + MS +BS	0.56 f	88.80	BS (T_5_–T_4_)	15.40
	T_6_: GCS + MS +PA	0.28 e	94.40	PA (T_6_–T_4_)	21.00
	T_7_: GCS + MS + BS + PA	0.09 e	98.20	BS + PA (T_7_–T_4_)	24.80

## Data Availability

The original contributions presented in this study are included in the article/Supplementary Materials. Further inquiries can be directed to the corresponding author(s).

## Supplementary Materials

The following supporting information can be downloaded at: https://www.mdpi.com/article/10.3390/toxics14070621/s1, Table S1: Method validation parameters for FMOC-derivatized glyphosate determination in soil, root, and leaf matrices using HPLC-UV; Table S2: Kinetic fitting parameters and first-order dissipation modeling of GLY in soil; Figure S1: Calibration curve of GLY analyzed by HPLC-UV after FMOC derivatization; Figure S2: HPLC chromatograms of FMOC–glyphosate (FMOC–GLY): (a) standard solution, (b) soil, (c) root, and (d) leaf.
