# Supplementary material for: Microbial-Assisted Phytoremediation of Glyphosate-Contaminated Soil by Medicago sativa: Biochemical and Detoxification Responses, Gene Expression, and Dissipation Kinetics
Source: Toxics. 2026 Jul 16;14(7):621. doi: 10.3390/toxics14070621 (PMC13417340; doi:10.3390/toxics14070621)
Supplement: Supplementary file 1 [file toxics-14-00621-s001.zip › toxics-4394927-supplementary.pdf]

Table S1. Method validation parameters for FMOC-derivatized glyphosate determination in soil, root, and leaf matrices using HPLC-UV

Matrix	Matrix effect (%)	Precision (RSD%, n=3)	Accuracy (Recovery %)
Soil	−1.74%	0.20-0.25	93.33–94.74
Root	+0.78%	0.18-0.22	89.05–91.69
Leaves	+1.57%	0.20-0.24	90.55–93.11

Table S2. Kinetic fitting parameters and first-order dissipation modeling of GLY in soil

Treatment	Regression equation	K (day^{-1})	Half-life $t_{1/2}$ (days)	R^2
GCSS	y=−0.00502x+a	0.01155	60	0.98
GCS	y=−0.00572x+a	0.01316	52.66	0.98
GCSS+MS	y=−0.00685x+a	0.01578	43.91	0.97
GCS+MS	y=−0.00908x+a	0.02092	33.12	0.99
GCS+MS+BS	y=−0.01454x+a	0.03349	20.69	0.98
GCS+MS+PA	y=−0.01879x+a	0.04328	16.01	0.98
GCS+MS+BS+PA	y=−0.0450x+a	0.1036	6.69	0.99

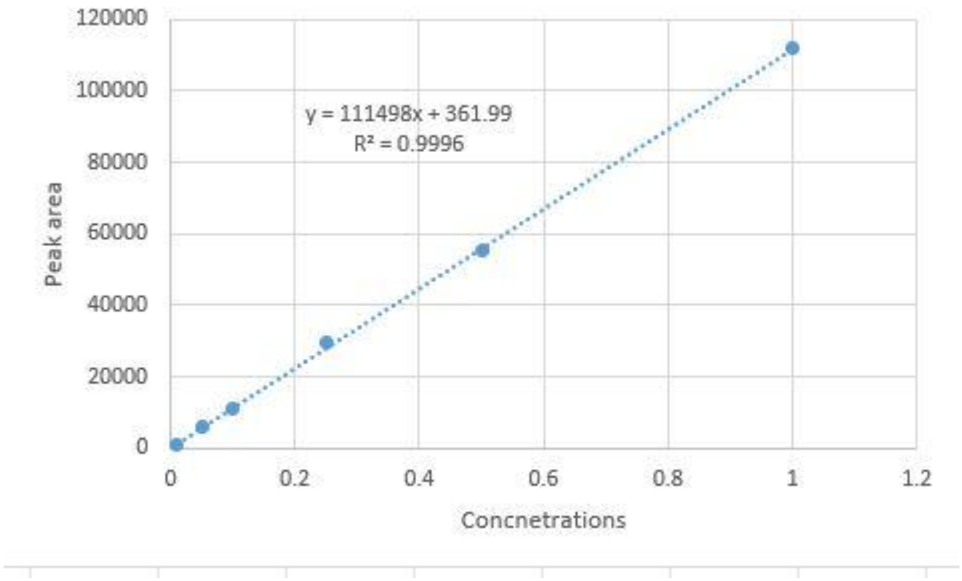

Fig. S1. Calibration curve of GLY analyzed by HPLC-UV after FMOC derivatization

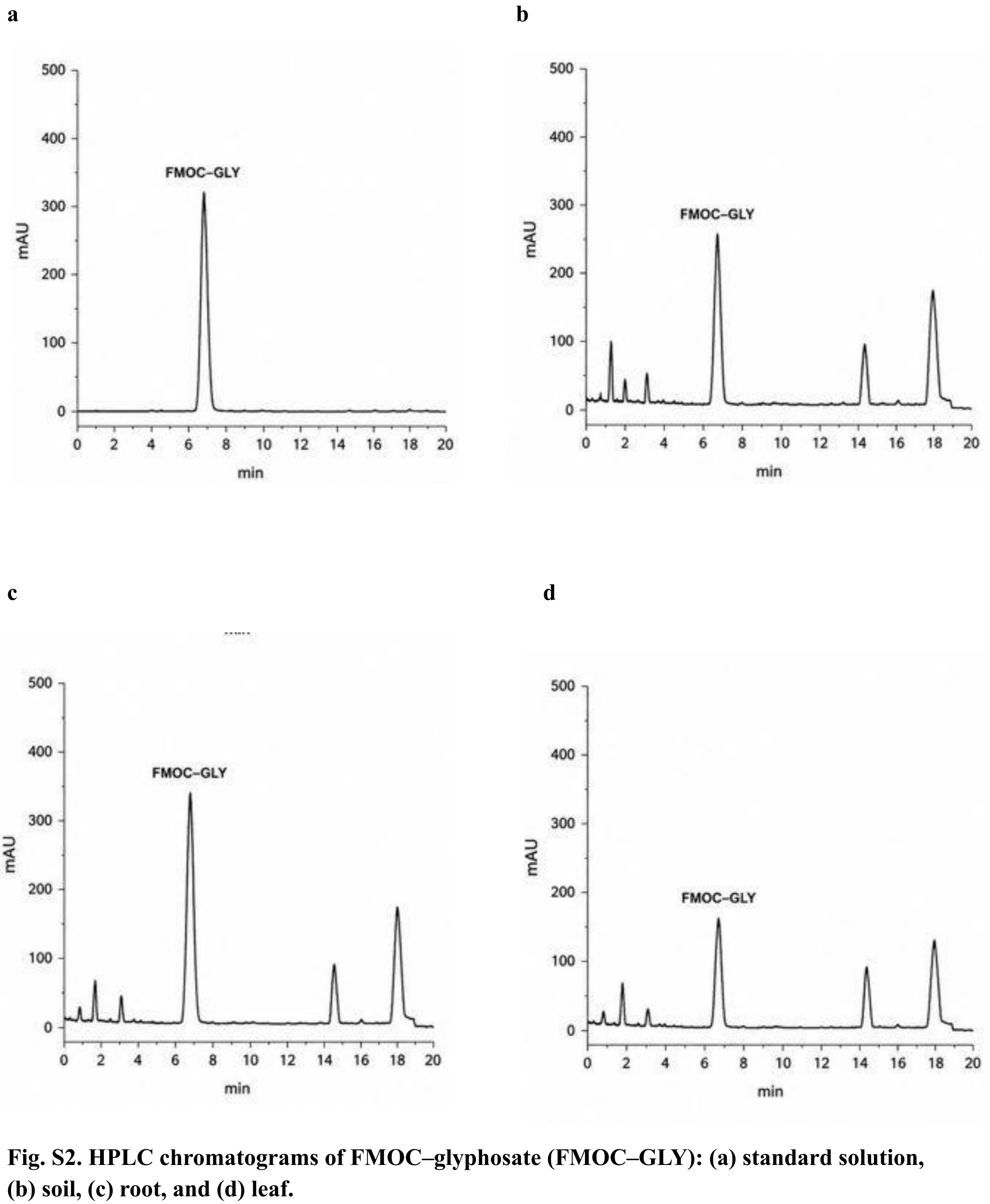

Fig. S2. HPLC chromatograms of FMOC–glyphosate (FMOC–GLY): (a) standard solution, (b) soil, (c) root, and (d) leaf.
